# Clinical evaluation of micro-fragmented adipose tissue as a treatment option for patients with meniscus tears with osteoarthritis: a prospective pilot study

**DOI:** 10.1007/s00264-020-04835-z

**Published:** 2020-10-07

**Authors:** Gerard A. Malanga, Paul S. Chirichella, Nathan S. Hogaboom, Teresa Capella

**Affiliations:** 1 New Jersey Regenerative Institute LLC, 197 Ridgedale Ave Suite 210, Cedar Knolls, NJ 07927 USA; 2 Department of Physical Medicine and Rehabilitation, New Jersey Medical School, Rutgers University, Newark, NJ USA; 3 Kessler Foundation, West Orange, NJ USA; 4 New Jersey Medical School, Rutgers University, Newark, NJ USA

**Keywords:** Meniscus, Knee osteoarthritis, Injections, Intra-articular, Adipose tissue, Knee injuries, Ultrasonography

## Abstract

**Purpose:**

The management of knee pain secondary to meniscal tears with osteoarthritis is limited by the poor inherent healing potential of the meniscus. Previous studies have reported on the benefit of autologous micro-fragmented fat as a therapeutic for various knee pathologies. The goal of this prospective pilot study was to determine the safety and potential treatment effect of micro-fragmented adipose tissue injection for patients with knee pain secondary to osteoarthritis and meniscal tears who have failed conservative management.

**Methods:**

Twenty subjects with knee pain secondary to osteoarthritis with associated meniscal tear after failed conservative management were enrolled in the study. Numeric Pain Scale (NPS) and Knee Injury and Osteoarthritis Outcome Scale (KOOS) following ultrasound-guided intra-meniscal and intra-articular micro-fragmented adipose tissue injections were examined at three, six and 12 months.

**Results:**

The mean NPS revealed a significant decrease in patient pain at the 1-year time point compared with baseline (5.45 to 2.21, *p* < .001). Similarly, overall, mean KOOS symptoms significantly improved from 57.7 to 78.2 (*p* < .001), with all 4 KOOS subscales demonstrating significant improvement at the final one year follow-up. One subject developed uncomplicated cellulitis at the harvest site which was treated with oral antibiotics. Other complications were minor and mostly limited to adipose harvest.

**Conclusion:**

This study demonstrated that micro-fragmented adipose tissue injected directly into a torn meniscus and knee joint using ultrasound guidance represents a safe and potentially efficacious treatment option for patients with knee pain suffering from degenerative arthritis and degenerative meniscal tears. A larger, randomized, controlled trial is warranted to determine efficacy.

**Trial registration:**

Clinicaltrials.org Identifier: NCT03714659

## Background

Meniscus tears are a common injury, with an estimated annual incidence as high as 60–70 per 100,000 [1]. Tears interfere with vital functions [2] of the meniscus and increase the risk of developing knee osteoarthritis [3]. Degenerative, as opposed to traumatic, meniscus tears are commonly found in association with osteoarthritis in patients over the age of 40. Depending on the severity of the tear and other indications, treatment typically starts with conservative methods (physical therapy, pain medications, etc.), then surgical methods, such as arthroscopic partial meniscectomy (APM), are recommended. The optimal management of these degenerative tears remains a challenge given the poor healing capacity of the inner, avascular portion of the meniscus where direct surgical repair techniques are commonly unsuccessful [4]. Unfortunately, APM has shown limited utility in improving knee pain and function in patients with degenerative meniscus tears when compared with exercise, especially in the setting of degenerative arthritis [5]. The lack of treatment alternatives for patients who have failed conservative management has increased the need for other non-operative treatments such as orthobiologics.

Orthobiologic treatments using platelet-rich plasma (PRP) injections of meniscus tears have been studied with mixed results. Mesenchymal stromal cell (MSC)–based therapies are potential candidates to meet the challenge of meniscal healing. MSCs act as trophic mediators by secreting a variety of cytokines and growth factors, which have been found to inhibit fibrosis and apoptosis, enhance angiogenesis, and stimulate the differentiation of tissue-intrinsic reparative or stem cells [6]. MSCs harvested from adipose [7] have been used for orthopaedic applications, and recent studies have demonstrated successful treatment of meniscal tears with adipose-derived stem cells [8].

Autologous, micro-fragmented adipose tissue (MFAT) may be harvested, processed, and transferred in a closed cylindrical system while preserving and enhancing the natural healing potential of adipose graft [9]. The process uses mild mechanical forces to both micro-fragment the adipose and wash away any pro-inflammatory oil and blood residues without the use of enzymes, additives, or separation centrifugation, while preserving the microarchitecture [10]. An essential part of that intact microarchitecture is the stromal vascular niche where pericytes are located. After injury or damage to the capillary wall, pericytes can detach and gradually convert into activated MSCs [11]. Although the exact mechanism of action is not fully understood, improvement in pain and function scores following intra-articular injection of MFAT has been reported in the setting of knee osteoarthritis and meniscal tear [12]. The rationale for considering MFAT injection over PRP and bone marrow aspirate is that the adipose tissue serves as a tissue filler in defects such as torn meniscus and cartilage lesions in addition to the noted potential anti-inflammatory and healing effects.

The aim of this pilot study was to determine the safety and potential treatment effect of MFAT as a treatment option for patients with knee pain secondary to osteoarthritis. After treatment with MFAT for knee pain, patients were assessed for procedure-related adverse events and for clinically meaningful changes in knee function and pain. The preliminary data from this study would ultimately be used in the development and management of a larger randomized controlled trial.

## Methods

### Study sample

This study received Institutional Review Board approval from the Institute of Regenerative and Cellular Medicine (LG-MN-201) and was registered with Clinicaltrials.org (Registration Number: NCT03714659). A total of 20 subjects were recruited to the study who were evaluated for knee pain at the clinic between January 2016 and January 2017. Inclusion criteria included age 35 and older; knee pain associated with symptoms of knee osteoarthritis or torn meniscus (i.e., localized joint line clicking, popping, giving way, pain with pivot or torque, episodic pain); joint line pain on palpation; subjective pain at the medial or lateral joint line for at least three months; MRI or arthroscopic evidence of meniscal tear; and failed conservative treatment. MRI evidence of meniscal tear was confirmed via community radiologists’ interpretation. Conservative treatment was defined as any combination of the use of anti-inflammatory or other medications for pain, physical therapy, corticosteroid injections, and/or hyaluronic acid (HA) injections. This also included patients who were told that they were a candidate for arthroscopic surgery by an orthopaedic surgeon due to the failure of conservative measures. Exclusion criteria included diffuse knee pain; chronically locked knee; history of prior knee surgery; assessment determining pathology requiring surgical management other than meniscal tear; treatment with PRP, cortisone (oral or injected), or HA injection within six weeks; malignancy within five years; or any disease or condition that may hinder or conflict with treatment. Though the criteria only required that the patients not receive any PRP, cortisone, or HA injections for six weeks prior to the study, no patients were previously treated with PRP or cortisone and no patient received HA injections within three months of the study, ensuring that any benefits from these injections did not affect study outcomes. Patients presenting with post-traumatic lesions of the meniscus were also excluded in order to maintain uniformity in the patient groups. Also considered were any contra-indications to lipo-aspiration such as history of bleeding disorder, infection, pregnancy, or allergies to anaesthetic agents.

All patients meeting inclusion criteria during the study period were invited to participate. Upon agreement and signing of an informed consent form, baseline demographic data were collected (i.e., age, sex, body mass index [BMI]). The type of meniscal tear and location was noted based on MRI results. Baseline Numerical Pain Scale (NPS) and Knee Injury and Osteoarthritis Outcome Score (KOOS) subscale scores were collected. Patients were then scheduled for a treatment date and were restricted from taking steroids or non-steroidal anti-inflammatory medications for three days prior to treatment. Patients were seen for initial follow-up at four weeks. They then completed surveys at three, six and 12 months, including the KOOS subscales and NPS.

The NPS is one of the most common measures of pain intensity and is used frequently in both research and clinical practice. The scale is well-validated and reliable for determining the change in pain over time [13]. Pain is rated on an 11-point scale, 0–10, with 0 representing “no pain” and 10 representing “worst imaginable pain.” It was well suited to this study given the ease of administration in person or via an online survey. The NPS minimum clinically important difference (MCID) for patients with knee pain secondary to osteoarthritis has been established as two points [14].

The KOOS is also widely used in research and clinical practice, including in large-scale databases and registries [15]. It is intended to be used in the setting of knee pain or injury that can result in post-traumatic osteoarthritis, including meniscus tears. It can be used over short- and long-term intervals to assess change over time. It consists of five subscales: pain, other symptoms, function in activities of daily living (ADL), function in sports and recreation (Sport/Rec), and knee-related quality of life (QOL). The re-test reliability has been established in patients with knee injuries [16] and the MCID has been determined as 8–10 [17]. We selected a change of greater than 10 on the KOOS as the MCID for this study. The primary endpoints of this study were changes in NPS and KOOS scores at 12 months.

### Surgical procedure

The treatment intervention consisted of percutaneous trephination of the meniscus tear and injection with MFAT into the tear and joint, which was harvested using the Lipogems® processing kit (Lipogems International SpA, Milan, Italy). This disposable kit allows for the aspiration, processing, and re-injection of autologous MFAT without the need for expansion or enzymatic treatment. Patients were placed supine on the procedure table and generally the abdomen was marked with a surgical marker in an oval, demarcating the region for fat harvest. In patients with low body fat or limited abdominal adipose, the lower lateral lower spine (i.e., “love handle”) area or posterolateral thigh was targeted. 500 cc of tumescent was prepared by combining 50 cc 1% Lidocaine with 1 cc of 1:1000 Epinephrine and sterile saline. After disinfecting the skin with ChloraPrep and bordering the area with sterile drapes, the tumescent was injected using an 18-gauge needle for local anesthesia. Next, a 17-gauge blunt cannula was inserted at the expanded entry point, irrigating the harvest site subcutaneously below Scarpa’s fascia with 60–120-cc tumescent. The Lipogems® kit was then assembled and connected to a bag of 1000 cc of sterile saline. The assembly is prefilled with saline solution and flushed by gravity into the waste bag to obtain a closed system free of air. After ten to 15 minutes, a 13-gauge blunt end cannula was then used to aspirate adipose tissue. The lipoaspirate was then injected into the device passing through a reduction filter, later allowing for draining of blood and oil residue into the waste bag. The central device containing stainless steel ball bearings is shaken for 30 seconds to further fragment and wash the lipoaspirate. When complete, the resulting MFAT is drawn into a syringe for injection.

The meniscal tear(s) was identified using a high-frequency linear ultrasound probe (Sonosite X-Porte; Fujifilm Sonosite, Bothell, WA, USA) in coordination with historical MR images. If a large effusion was detected, this was aspirated prior to MFAT injection. Utilizing sterile technique, MFAT was injected under direct ultrasound guidance into the hypoechoic defects using primarily an 18-gauge 3-inch needle attached to a 3-mL syringe within the visualized meniscal tears and knee joint. We used a 22 gauge 1.5-inch needle when needed due to joint space narrowing. A trephination technique was used to direct the needle into the meniscus from an outer to inner approach as described by Baria et al. [18]. This allowed for lipofilling of the soft tissue defects of the meniscus using 1–2 mL of MFAT into the tear. The remaining available MFAT was then injected into the knee joint using a lateral suprapatellar approach, under ultrasound guidance. Per request, four subjects received a contralateral knee MFAT injection without a confirmed diagnosis of meniscal tear. Outcomes data referred only to the knees originally enrolled in the study.

Post-injection guidelines including weight bearing restrictions—non-weight bearing with crutches for one week, then weight bearing as tolerated for simple daily activities—while refraining from running and jumping activities and repetitive flexion beyond 90 degrees for four weeks total. If there was no significant pain, swelling, or joint line tenderness, and near full range of motion, unrestricted activities were allowed at the six to eight week timeframe. Complications of both the harvest and injection sites were recorded via questionnaire at one and four week follow-ups. Patients were restricted from taking non-steroidal anti-inflammatory medications or steroids for two to three weeks after the procedure, but were allowed two to three days of either tramadol or oxycodone for post-procedural pain.

### Statistical analysis

Significance was set to *p* < .05 for all tests, which were conducted using SPSS v.21 (IBM, Inc., Armonk, NY). Linear mixed-effects models were employed to evaluate changes in KOOS subscale and NPS scores at each time point. Outcome variables (KOOS, NPS scores) were specified at level 1 and individuals were specified at level 2. Time was treated as a fixed effect, while the intercept was treated as a random effect to account for variance in baseline scores. Using these models, changes in outcomes could be examined within individuals over time while accounting for missing data and disparate lengths in time between each point of data collection [19]. Post hoc analyses with Bonferroni corrections were conducted to determine how outcomes differed at each time point compared with baseline.

## Results

Twenty individuals were recruited for the study. Average age and BMI were 59.8 ± 6.5 and 28.6 ± 4.8, respectively. Eleven participants were male. One male subject injured his contralateral, untreated knee following the procedure requiring surgical intervention and thus was excluded from the study. Of the remaining 19 subjects, three underwent injections into meniscal tears bilaterally. Demographic and clinical data are summarized in Table 1. Notably, tears were most commonly found in the medial compartment (82.6%). Complex tears were most common with a prevalence of 73.9%. Only two patients were documented as not having evidence of osteoarthritis on MRI, with most of the cases being graded as mild to moderate in severity.

**Table 1:** Characteristics of those treated with micro-fragmented adipose tissue for meniscal tears, including demographic and clinical information

Clinical variable		Frequency (%) or mean ± SD
Meniscal tear location (*n* = 23^a^)	Medial	19 (82.6%)
	Lateral	3 (13.0%)
	Both	1 (4.3%)
Tear type (MRI) (*n* = 23^a^)	Vertical	2 (8.7%)
	Radial/oblique	3 (13.0%)
	Horizontal	1 (4.3%)
	Complex	17 (73.9%)
Osteoarthritis grading (MRI)	None	2 (8.7%)
	Mild	7 (30.4%)
	Moderate	13 (56.5%)
	Severe	1 (4.3%)
Knee injected (*n* = 23^a^)	Right	6 (26.1%)
	Left	11(47.8%)
	Bilateral	3 (13.0%)
Total injectate volume (mL)		7.6 ± 2.3

*Notes*. Continuous variables are presented as means ± standard deviations (ranges), while categorical variables are presented as frequencies (percentages)

^a^23 knees were injected among the 20 participants

There were no reported serious adverse events reported during or following the study. One patient reported local erythema and swelling at the harvest site without constitutional symptoms. The decision was made to treat as an uncomplicated cellulitis with amoxicillin/clavulanate 875–125 mg twice daily for ten days with complete resolution of symptoms. Minor complications included soreness at the harvest site in ten of the subjects (52.5%), and reports of “haematoma” formation in three patients (Table 2). Three patients complained of swelling in the injected knee after the procedure though was self-limited. All reported minor complications resolved within four weeks following treatment with the vast majority without complaints/limitations after one week following treatment.

**Table 2:** Reported complications (*N* = 20) after treatment with micro-fragmented adipose tissue

Harvest site		Injection site	
Bruising	2	Swelling	3
Bleeding	1	Soreness	0
Hematoma	3	Bruising	0
Drainage	1	Infection	0
Infection	1		
Soreness	10		

**Fig. 1:**
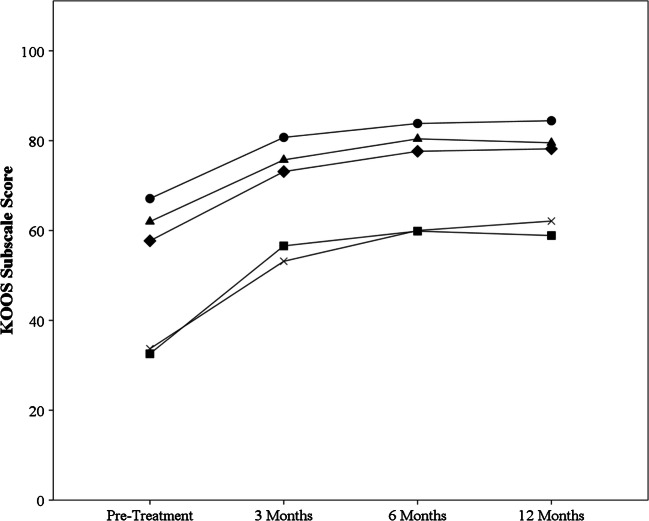
Changes in Knee Injury and Osteoarthritis Outcome Score (KOOS) subscale scores in response to treatment. Increases were seen across all five subscales, which leveled off after 6 months through to 12 months post-treatment. Triangle = symptoms subscale, circle = pain subscale, square = activities of daily living subscale, diamond = sports and recreation subscale, “X” = quality of life subscale

**Fig. 2:**
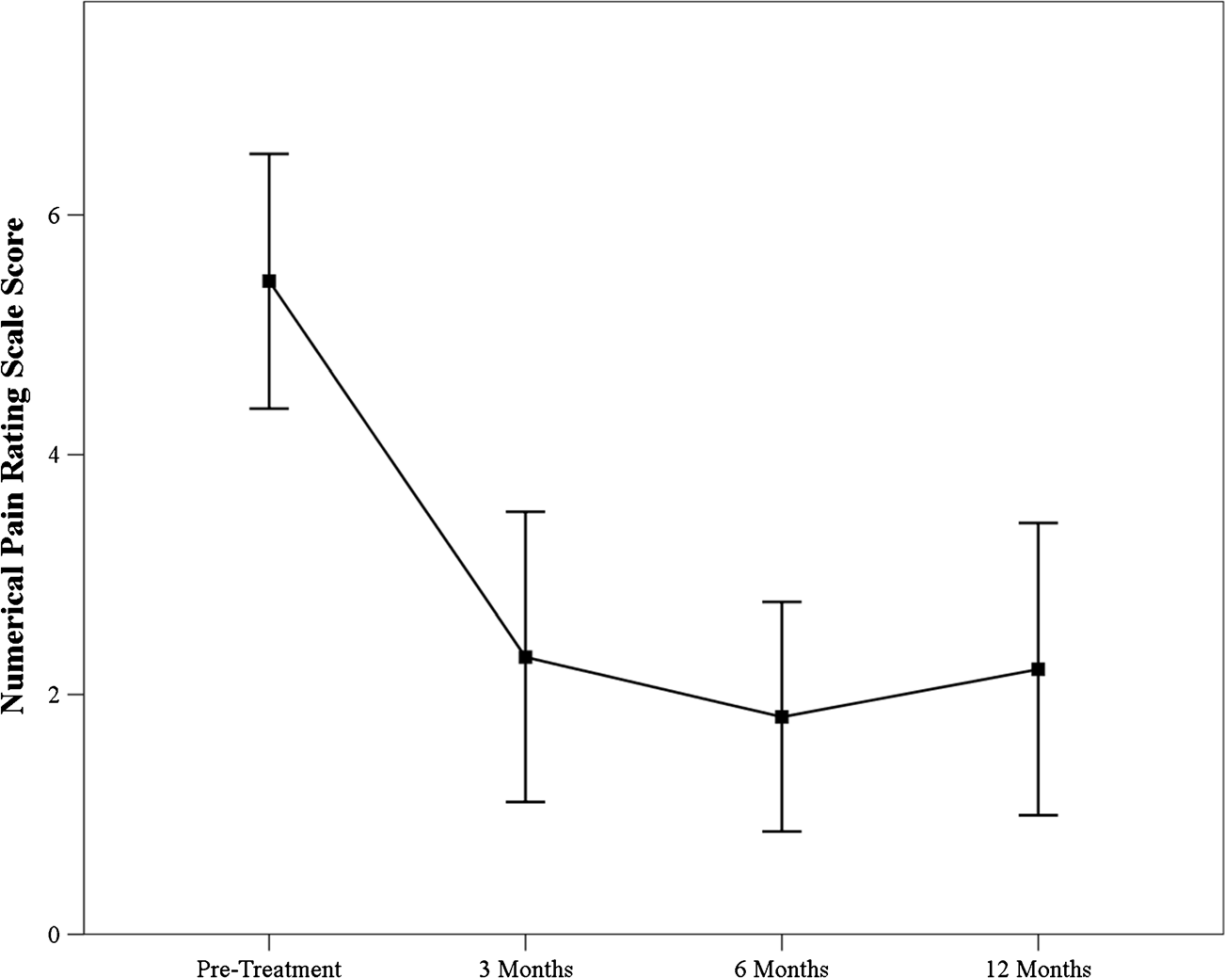
Overall changes in numerical pain scale scores over the duration of the study. Decreases were observed at all time points were observed relative to pre-treatment pain levels. Improvements in pain appeared to level and remain steady after 6 months

Nineteen subjects completed the 12-month assessment. Three were unavailable for the three month assessment, and four did not complete the six month assessment. Statistically significant improvements in all KOOS subscale and NPS scores were noted at all time points with respect to baseline (*p* < .01; Table 3). No other significant differences were observed (*p* > .05). Temporal changes are presented graphically in Figs. 1 and 2.

**Table 3:** Knee Osteoarthritis and Injury Outcome Score and Numerical Pain Scale subscale scores at each time point between baseline and 12 months post-treatment

Measure	Baseline (*n* = 19)	3 months (*n* = 16)	6 months (*n* = 15)	12 months (*n* = 19)	% ≥ MCID
NPS	5.45 ± 2.2	2.58 ± 2.3*	1.95 ± 2.0*	2.21 ± 2.5*	78.9
KOOS Symptoms	57.7 ± 15.4	73.1 ± 16.8*	77.6 ± 15.5*	78.2 ± 17.4*	63.2
KOOS pain	62.0 ± 17.3	75.7 ± 15.9*	80.4 ± 15.9*	79.5 ± 19.0*	57.9
KOOS ADLs	67.1 ± 17.5	80.7 ± 14.6*	83.8 ± 15.5*	84.4 ± 17.8*	57.9
KOOS sports/rec	33.7 ± 24.4	53.2 ± 30.3*	60.0 ± 30.0*	62.1 ± 31.2*	73.7
KOOS QOL	32.6 ± 23.3	56.6 ± 26.7*	59.9 ± 28.0*	58.9 ± 31.1*	73.7

*Notes*. Results are presented as means ± standard deviations and average percentage changes, with the relevant sample size at each time point. Percentages of participants with changes greater than or equal to MCID for each respective scale at 12 months are also presented. *NPS*, Numerical Pain Scale; *KOOS*, Knee Osteoarthritis and Injury Outcome Score; *ADLs*, activities of daily living subscale; *QOL*, quality of life subscale; *NPS*, numerical pain scale; *MCID*, minimal clinically important difference

*Significant difference with respect to baseline (*p* < .01)

## Discussion

Despite a growing body of evidence to recommend against APM in the setting of degenerative changes in the knee [5], many patients continue to opt for surgery due to a perceived lack of available treatment options. And after the eventually perceived failure of conservative management and progression of osteoarthritis, patients elect for total knee arthroplasty at higher rates and younger ages [20]. As meniscus tear management shifts to preservation and repair, it is clear that regenerative treatments will play a role in counteracting the menisci’s poor intrinsic ability to heal, especially in the setting of knee osteoarthritis. The present pilot study demonstrates that MFAT represents a safe and potentially efficacious treatment option for degenerative meniscus tears and may be used to help guide further studies.

A variety of regenerative medicine treatments have been explored as options to treat both meniscal tears and knee osteoarthritis such as platelet-rich plasma, bone marrow aspirate concentrate (BMAC), and adipose-derived mesenchymal stem cells. MFAT was chosen for this study due to distinctive advantages over BMAC and stromal vascular fraction (SVF) in ease of obtaining stem cells from a patient without the need for multiple visits, enzymatic digestion, or cell expansion [21]. Recent research comparing MFAT with SVF demonstrated enhanced growth factor secretion in MFAT which is attributed to an intact perivascular niche [22]. Additionally, MFAT made through mechanical processing and washing of adipose tissue qualifies as “minimal manipulation” under the Food and Drug Administration (FDA) guidelines in the USA unlike enzymatic processing used in SVF [23]. The utilization of enzymes to assist in adipose cell separation requires an FDA-approved Biologic License Application [24] partially limiting clinical applicability.

One important aspect of our study is the use of direct ultrasound visualization and lipofilling of meniscal defects rather than only performing an intra-articular injection. There is a considerable amount of literature regarding the use of adipose grafting in the fields of reconstructive or cosmetic surgery for the filling and supporting of tissue defects [25–27] in addition to its innate regenerative capacity. We utilized a technique for performing intra-meniscal injections under ultrasound guidance, which was described and validated by Baria et al. [18], in an attempt to accurately place MFAT within meniscal tears and hopefully maximize the treatment effect.

An alternative orthobiologic treatment for meniscus tears and osteoarthritis is injection of platelet-rich plasma (PRP). The basis of this treatment is the autologous source of platelets which release multiple products with important roles in healing, including cytokines, chemokines, and more than 1500 growth factors [28–30]. In vitro studies indicate that exposure to anabolic cytokines, such as PDGF-AB which is released from PRP, enables fibrochondrocytes within the avascular region of the meniscus to proliferate and form new matrix [31]. Clinical trials also support the benefit of PRP in meniscal and osteoarthritic indications, though the benefits seen are often minimal [32, 33]. Similarly, a double-blind RCT was conducted to compare outcomes of meniscus trephination with or without PRP injection; failure (defined as meniscus non-union observed in the magnetic resonance arthrography or arthroscopy) was noted for 70% of patients in the control group, while only 48% failure was noted in the PRP group. There was also greater symptom improvement and less eventual APM in the PRP group [34]. The minimal benefits generally observed in the use of PRP to treat osteoarthritis and meniscus tears discouraged its use in this study.

As a group, the study sample showed clinically meaningful improvement in pain, function, and QOL measures despite having previously failed other treatments for their knee pain. These results were similar to two published case reports where individuals with history of knee pain following meniscus tear in the setting of degenerative osteoarthritis were treated with ultrasound-guided intra-meniscal MFAT injections [12, 35]. All subjects avoided the need for APM for their knee pain during the study period. This may be compared with the cross-over rates from conservative management to APM in the Katz study as high as 21–30% at six to 14 months in various randomized controlled trials [36]. Furthermore, no participants reported serious adverse events after the procedure, which is consistent with other studies evaluating the effects of intra-articular MFAT [12, 35].

While the results are encouraging, this study does have several important limitations. This study was designed as a prospective cohort pilot study and no control group was utilized; thus, no definitive conclusions can be drawn regarding efficacy. As a result of this limitation, it is not known whether the treatment effect was caused by injection into the meniscus or into the intra-articular space, or a combination of both. As well, percutaneous trephination of the meniscus with normal saline and PRP has shown some treatment effect for horizontal tears [34] and thus may have contributed to the treatment effect observed in the present study. To mediate this limitation in a future study, a control group of individuals who solely receive intra-articular injections without injection into the meniscus and another which receives trephination of the meniscus with normal saline could be included. This study also lacked strict classification of the meniscus tears, and in future studies, a verifiable classification system should be used. Inclusion of more quantitative measures of tissue healing should also be considered. One example is measuring meniscus volume using quantitative MRI [37], which has been utilized in the past to demonstrate cartilage and meniscal growth following a bone marrow–derived aspirate injection [38]. Including these objective measures would yield a better understanding of the physiological effect of MFAT on meniscal healing. It should be noted that the majority of arthroscopic meniscus surgery research does not include any post-operative imaging such as MRI. Another quantitative measure that could be utilized is analgesic drug consumption in the months following the procedure (not the immediate post-operative period); as patients often use these types of drugs to relieve pain, a reduction in the use of pain-killers can be a tool to evaluate the efficacy of the treatment.

Additionally, the sample size was too small for subgroup analyses. Considering the potential impact of age and gender on meniscus tears and healing, the inclusion of these analyses could yield important clinical information. Participants were not blinded to the treatment, risking treatment bias. The placebo effect of having a knee injection has been well studied in the setting of osteoarthritis [39] and may have contributed to the outcomes. Due to the nature of the procedure (i.e., fat harvest), it is difficult to blind participants to treatment aside from conducting a sham study, which raises ethical concerns. Ameliorating these potential sources of bias would likely yield a strong study to determine efficacy. To better determine the effectiveness of this treatment modality specifically for meniscus tears, a randomized, controlled study on meniscus tears in 20–40-year-old subjects has been initiated Clinical trial # NCT04274543. Furthermore, three of the subjects received bilateral knee MFAT injections which mutually influenced change in KOOS which cannot be only attributed to the index knee.

## Conclusion

**Fig. 3:**
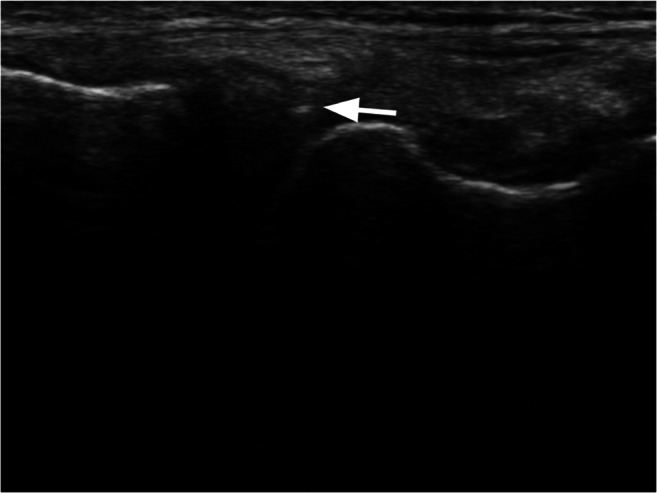
Needle tip visualized in an out-of-plane view for MFAT injection into the meniscus under ultrasound guidance

This study demonstrated that MFAT injected directly into meniscal tears following trephination of the tear along with a joint injection under direct ultrasound guidance represents a safe and clinically significant treatment option for patients with degenerative meniscus tears and knee osteoarthritis (Fig. 3). Additionally, the study suggests that improvement in knee pain and function scores may be sustained for up to one year. This information can help guide the development of a larger, randomized, controlled trial to determine the efficacy of MFAT. Inclusion of pre- and post-surgical MRI and other quantitative outcomes should be considered, as well as the recruitment of a large and diverse sample, to better understand physiological treatment effects.
